# Immune System Disequilibrium—Neutrophils, Their Extracellular Traps, and COVID-19-Induced Sepsis

**DOI:** 10.3389/fmed.2021.711397

**Published:** 2021-08-18

**Authors:** Colm Keane, Matthew Coalter, Ignacio Martin-Loeches

**Affiliations:** ^1^Department of Anaesthesia and Intensive Care, St. James's Hospital, Dublin, Ireland; ^2^Multidisciplinary Intensive Care Research Organization (MICRO), Trinity College Dublin, Dublin, Ireland

**Keywords:** neutrophil, neutrophil extracellular trap, COVID-19, NETosis, immune system, innate, SARS-CoV-2

## Abstract

Equilibrium within the immune system can often determine the fate of its host. Severe acute respiratory syndrome coronavirus 2 (SARS-CoV-2) is the pathogen responsible for the coronavirus disease 2019 (COVID-19) pandemic. Immune dysregulation remains one of the main pathophysiological components of SARS-CoV-2-associated organ injury, with over-activation of the innate immune system, and induced apoptosis of adaptive immune cells. Here, we provide an overview of the innate immune system, both in general and relating to COVID-19. We specifically discuss “NETosis,” the process of neutrophil release of their extracellular traps, which may be a more recently described form of cell death that is different from apoptosis, and how this may propagate organ dysfunction in COVID-19. We complete this review by discussing Stem Cell Therapies in COVID-19 and emerging COVID-19 phenotypes, which may allow for more targeted therapy in the future. Finally, we consider the array of potential therapeutic targets in COVID-19, and associated therapeutics.

## Introduction

Equilibrium within the immune system can often determine the fate of its host. In sepsis, and many other inflammatory syndromes, the host's immune system performs a balancing act between the protection it offers through eradication of the offending pathogen, vs. the constant threat of an immune-mediated pathophysiological maelstrom.

Severe acute respiratory syndrome coronavirus 2 (SARS-CoV-2) is the pathogen responsible for the coronavirus disease 2019 (COVID-19) pandemic (1).

**Figure 1:**
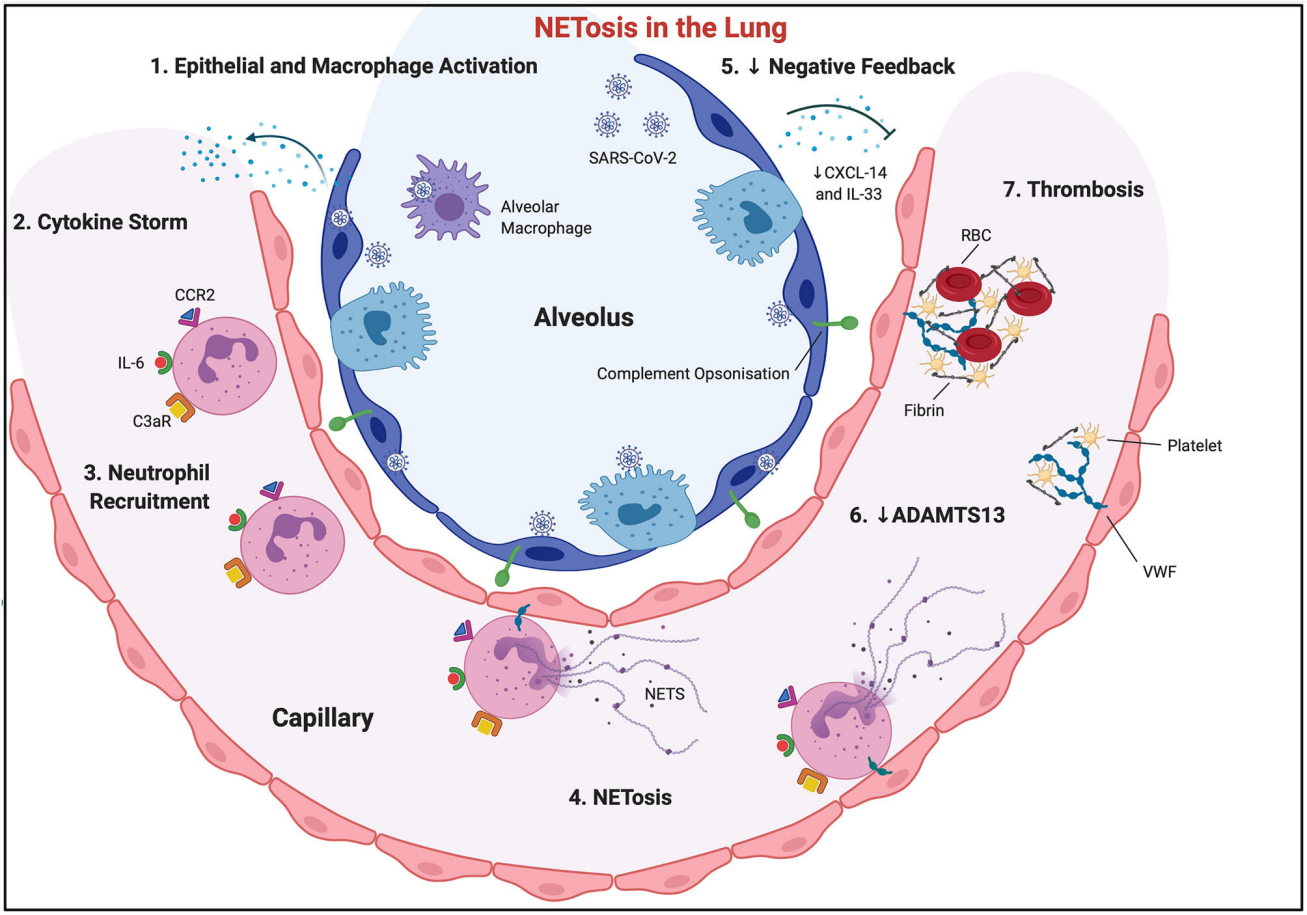
NETosis in the lung. SARS-CoV-2 virus invades alveolar epithelial cells, activating them, along with local macrophage populations (1). This causes a surge in the production and systemic release of cytokines and chemokines (cytokine storm) (2), with subsequent neutrophil recruitment (3). Activated endothelial cells exocytose Von Willebrand Factor, which allows neutrophil and neutrophil extracellular trap adherence to the vascular wall. CCL2 and IL-6 encourage “NETosis” (4). Negative feedback loops may be inhibited, through reduced CXCL-14 and IL-33, allowing sustained and enhanced immune cell recruitment (5). Complement C3 is also released from NETs, further propagating “NETosis,” and allowing opsonisation of surrounding tissue, which will ultimately necrose. NETs downregulate ADAMTS13, allowing VWF multimer development (6). Subsequent fibrinogen and platelet trapping occurs, which, along with red blood cells, encourages fibrin cross-linking, and ultimate vessel thrombosis.

SARS-CoV-2 breaches the alveolar epithelial membrane after binding to the human angiotensin-converting enzyme (ACE) 2 receptor. Subsequent viral RNAs serve as pathogen-associated molecular patterns (PAMPs), which are then sensed by Toll-like receptors (TLRs) (2). This results in epithelial cell activation, initiating a cascade of innate immune cell chemoattraction (Figure 1) (3). This immune cell infiltration causes acute respiratory distress syndrome (ARDS) locally in the lungs, and septic shock, coagulation dysfunction, and multiple organ dysfunction syndrome beyond the lungs (2). The mechanisms behind this distal organ injury are multiple, but immune dysregulation remains one of the main pathophysiological aetiologies. Neutrophil migration is affected with SARS-CoV-2 sepsis.

This review will focus on the innate immune system in COVID-19-induced sepsis and subsequently discusses stem cell therapies, emerging COVID-19 phenotypes and potential therapeutic targets.

## The Innate Immune System

Once a pathogen enters the body, the innate immune system must recognise this as foreign and initiate an immune response, with a view to the pathogen's destruction or elimination. Cells of innate immunity originate largely from the common myeloid progenitor cells in the bone marrow before differentiating into cells such as macrophages, dendritic cells, and granulocytes, including neutrophils (4). These cells, amongst others, recognise PAMPs, which are then sensed by pathogen recognition receptors (PRRs) such as TLRs (2). This discriminates non-self from self and allows for phagocytosis, degradation and pro-inflammatory cytokine signalling to alert cells downstream to the invader.

One of the major weapons of the innate immune response is the macrophage, differentiated from the monocyte (5). Macrophages have a role in immune surveillance, phagocytosis of pathogens and clearance of cell debris or apoptotic cells (efferocytosis), as well as tissue remodelling after insult (6). They are activated through PAMPs or self-derived damage-associated molecular patterns (DAMPs) binding to PRRs like TLRs, NOD-like receptors (NLR), and RIG-I-like helicases. Macrophages then initiate signal transduction pathways, *via* mediators like myeloid differentiation primary response 88 (MyD88), that culminate in the production of pro-inflammatory cytokines and chemokines (7). Macrophages can be broadly separated into two opposing phenotypes, pro-inflammatory (M1) and anti-inflammatory (M2) (8). Originally, macrophages were thought to share their monocyte precursor with dendritic cells, displaying different cell surface markers like CD11b which aid their primary functions (6). However, more recent findings challenge this and suggest a lymphoid origin for dendritic cells (9). Dendritic cells (marked by CD11c) specialise in antigen presentation *via* major histocompatibility complex (MHC) molecules and serve as a link between the innate and adaptive immune system, recruiting lymphocytes (10).

Neutrophil maturation in the bone marrow, under the regulation of granulocyte colony stimulating factor (G-CSF), results in circulating short-lived mature neutrophils. PAMPs in infected tissue bind to PRRs, initiating a cascade of events, generating chemotactic, and haplotactic gradients (e.g., CXCL-2) that recruit activated neutrophils to the affected area (11). M2-like macrophages increase targeted neutrophil recruitment to injured tissue *via* CXCL-2 secretion. Corresponding CXCR-2 receptors on neutrophils bind CXCL-2, and appropriate transendothelial neutrophil migration occurs to the injured tissue (12). Once at the designated tissue, neutrophils have a variety of anti-microbial effector functions like phagocytosis, degranulation of toxic substances such as nitric oxide and reactive oxygen species, and the release of neutrophil extracellular traps (NETs) (11). Elimination of the invading organism can then successfully be achieved (13).

## The Complement System

Another component of the innate immune system is the complement system. It is an auxiliary defence mechanism of innate immunity. It was discovered in 1896 by Bordet and named for its ability to “complement” antibodies in their antimicrobial defence (14). It comprises of over 30 soluble serum proteins, mostly proteases, which are cleaved and activated in sequence to elicit an effect. Low-level complement system activity maintains homeostasis, with ability for rapid activation in response to trauma or infectious insults (15). Cellular invasion by SARS-CoV-2, and the subsequent “cytokine storm” results in an excessive and unsustainable complement system activation (16), with C3 activation resulting in the production of proinflammatory mediators and opsonisation of the pathogen, and the formation of the membrane attack complex (MAC) made up of C5–C9 (14).

Three pathways exist—the classical, lectin, and alternative pathways. They differ in their initial steps, with the classical pathway requiring C1q and an antibody-antigen interaction (17). The lectin pathway is immunoglobulin-independent, using PRRs like mannose-binding lectin to recognise foreign molecules (17). The alternative pathway is continuously activated by spontaneous hydrolysis of C3 and can be upregulated by bacterial endotoxins, yeasts and immunoglobulins (18). The pathways converge on C3 convertases, resulting in the production of proinflammatory mediators, opsonisation of the pathogen's surface with markers such as C3b and lastly, the formation of the membrane attack complex (MAC) made up of C5–C9 (14). The MAC inserts into the lipid bilayer, allowing the dysregulated transmembrane movement of water and ions and subsequent lysis of the target cell.

In COVID-19 infection, JAK-STAT signalling induces the expression of C3 and Factor B resulting in alternative pathway activation, and intracellular processing of complement proteins (19), while in the extracellular space SARS-CoV-2 activates the lectin pathway (20). Complement hyperactivation is key to the detrimental effects of COVID-19, shown in two recent studies where higher complement activation products correlated with increased disease severity (19, 21). Factor D, upregulated by COVID-19 and involved in the alternative pathway, is correlated with markers of endothelial cell injury (e.g., angiotensin 2) and coagulation (e.g., vWF), possibly contributing to the association between COVID-19 and coagulopathy (21). Potential therapeutic mechanisms to reduce or prevent complement-mediated damage in COVID-19 are discussed below.

## Sepsis and COVID-19 Crosstalk

There has been much advancement in the understanding of the host response to infectious disease in the last decade. It is now well accepted that the mechanisms of damage of pathogens are not limited to their direct virulence, but also the host's immune response to the pathogen. These secondary reactions can range from localised to systemic, and manifest in the form of sepsis — “a severe, potentially fatal, organic dysfunction caused by an inadequate or dysregulated host response to infection (sepsis-3)” (22). There were 48.9 million cases of sepsis worldwide in 2017, accounting for 20% of all deaths (23), marking this as an extremely important disease to better understand and manage.

The emergence of SARS-CoV-2 has dramatically changed the landscape of communicable disease. The most common causes of death in these patients are sepsis and respiratory failure. A relatively new phenomenon of viral sepsis is being widely seen (24) and is similar to the well-characterised bacterial sepsis in the literature. Scientific efforts are underway to understand the disease's effects on the body and immune system to repurpose and develop therapies to improve outcomes and save lives. The overlap between COVID-19 and sepsis for individual aspects of innate immunity is discussed below.

### Cytokine Storm

Sepsis is a complex combination of various dysregulated immune response mechanisms. The cytokine storm occurs in the early phase (hours to days) of sepsis where PAMPs are recognised by PRRs on innate immune cells causing a “hyper-inflammatory” innate immune response (25). Influenza, a disease similar to SARS-CoV-2, was the first infectious disease where the cytokine storm was characterised in 2003 (26). Activated PRRs initiate signalling pathways, resulting in the production of proinflammatory cytokines like TNF-α, IL-1β, interferon regulatory factor 3 (IRF3), IRF7, or adaptor-protein 1 (AP-1), under the regulation of the transcription factor NF-κB (27). The activation of PRRs by SARS-COV-2 viral RNA (specifically TLR3, TLR7, TLR8, and TLR9) results in epithelial cell activation, and the production of numerous proinflammatory molecules including TNF-α, IL-1α, IL-1β, IL-2, IL-6, IL-8 (CXCL-8), IFN-γ, and CCL-2 (Figure 1) (2). This cytokine milieu is involved in ARDS pathological propagation in COVID-19 populations (3, 28).

The result is the increased activation, proliferation, or migration of immune cells (Figure 1). In sepsis, PRR expression is dysregulated with higher levels of TLR4 mRNA and TLR2 receptors (29). Levels of IL-1β, amongst other pro-inflammatory cytokines, were found to be higher in patients who died of sepsis than in those who survived (30). Similarly, IL-6 overexpression has been associated with more severe sepsis and worse outcomes (31), potentially due to complement activation (32). In COVID-19, IL-6 becomes upregulated (TLR-8-induced in neutrophils, C5a-induced in monocytes/macrophages), enhancing neutrophil superoxide production, and delaying apoptosis (3). IL-6 production is thought to be a major initiator of the “cytokine storm” in COVID-19 (2) leading to repeated attempts to modulate IL-6 activity in sepsis, and more recently in COVID-19, with varying success (33, 34).

Cell death, caused by microbes as well as the host inflammatory response, releases endogenous DAMPs, further activating PRRs, auto-amplifying the cytokine storm (35) and initiating a cascade of innate immune cell chemoattraction (3). Chemokines also play a major role in immune cell chemoattraction in sepsis. They are small molecules specialised in the recruitment of leukocytes and their release from the bone marrow or spleen. C-X-C chemokine secretion from tissue-resident macrophages is also upregulated in COVID-19. Recruited neutrophils release CCL20, which *via* CCL6 attracts dendritic cells, memory T and B cells, and macrophages to the site of inflammation. A lack of chemokines, or their receptors, leads to an immunosuppressed state where the host is more susceptible to sepsis-induced death (36). Conversely, CXCL-14 potently inhibits epithelial cell chemotaxis, and is downregulated in COVID-19 allowing sustained and enhanced immune cell recruitment (3).

As detailed above, SARS-CoV-2 breaches the alveolar epithelial membrane after binding to the human ACE-2 receptor. The protein transmembrane protease serine 2 (TMPRSS2) is an essential facilitator of SARS-CoV-2 viral cell entry, in conjunction with ACE-2 (37). The SARS-CoV-2 spike protein subsequently interacts with ACE-2, downregulating it (38). Without ACE2, angiotensin-II concentrations and signalling potential increase, upregulating the activation of inflammatory pathways in epithelial and endothelial cells, particularly the p38/MAPK pathway (39). This intracellular inflammatory upregulation, combined with a downregulation of cytokine-release checkpoints (CXCL-14) contributes to the “Cytokine Release Syndrome” which is now well-described in COVID-19, and likely highly pathological. As described in a prior review (40), it is established that the severity of sepsis may be more linked to the host's response to the pathogen, rather than the virulence. Another study discusses the link between clinical manifestations and host gene transcription patterns in staphylococcal infection (41), and noted a significant link between pattern of cytokine gene expression and disease severity, regardless of the causative pathogen (42). This could be relevant in the study of COVID-19, where researchers hypothesise how a single pathogen can have such a varied effect on different individuals ranging from asymptomatic to devastating ARDS and multi-organ dysfunction syndrome (MODS).

### The Inflammasome

The importance of the NOD-like receptor pyrin containing-domain 3 (NLRP3) inflammasome is becoming better understood in sepsis. This macromolecular protein complex converts pro-caspases to their mature form, inducing the release of pro-inflammatory cytokines like IL-1β (43). In sepsis, activation of TLRs primes the inflammasome through NF-κB, and it is activated by ROS release and mitochondrial damage by phagocytic cells, having a widespread effect on various systems (44). It is active in patients with COVID-19 and higher levels of IL-18 and Casp1p20 are correlated with COVID-19 severity and poor clinical outcome (45).

### Mitochondria

Mitochondria also have a pivotal role in sepsis, well beyond their classical role in oxidative phosphorylation and ATP production. Research has shown sepsis-induced mitochondrial dysfunction may play a pathophysiological role in major organ dysfunction and death (46). For example, in sepsis, mitochondria increase free radical production, propagating the cytokine storm from Kupffer cells in the liver (47), and inducing caspase-mediated apoptosis in the heart causing cardiac dysfunction (48). There is also a dysregulated electron transport chain that may cause a rise in lactate (49). There is evidence of reduced mitochondrial gene expression in individuals who die of sepsis, signifying a loss of function in mitochondria (50). In COVID-19, there is widespread mitochondrial dysfunction caused by inflammation, cytokine storm, oxidative stress, microbiota dysregulation, iron overload, and ROS accumulation (51).

### Immunosuppression

Equilibrium of the host's immune response to an offending pathogen is important. A balance must be struck between pro- and anti-inflammatory responses to effectively create an immune response to recognise and eliminate the microbial threat and prevent secondary infection, without excess damage to host cells and organs and to allow full resolution of inflammation. If a patient survives the initial cytokine storm, long-lasting immunosuppression may increase the complications of secondary infection, potentially leading to their death. It has been reported in the literature, agreeing with our clinical observations, that 15% of hospitalised COVID-19 patients, and 50% of those who subsequently die, acquire a secondary infection (52). The incidence of ventilatory-associated lower respiratory tract infections in SARS-CoV-2 patients is significantly higher than in patients with influenza (53). This is likely also the case for COVID-19 associated pulmonary aspergillosis (54). Through genome-wide transcription profiling, it has been possible to quantify downregulation of antigen presentation and suppression of T cell activation to a much greater degree in those who died from sepsis (55). We also know that much mechanistic immunological research has demonstrated that an intact T cell mediated immune response is required for eliminating and suppressing viral infections (56).

Lymphopenia has consistently correlated with disease severity throughout COVID-19. It is rare in children, in whom COVID-19 mortality is very low, and much more common in the elderly, where higher mortality rates are seen (57). It is also seen that there is a consistent and marked reduction in T cell counts, which is not always the case with B cell counts (58), which may question the necessity of B cell involvement in mounting a successful response to COVID-19.

Several possible mechanisms exist for this lymphocyte depletion in COVID-19. The cytokine release syndrome, detailed above, especially cytokines IL-6 and TNF-α, may lead to massive lymphocyte death. Regulatory T cells seem to be spared however. These cytokines may also reduce the toxicity of T cells and NK cells (59). COVID-19 can also result in T cell exhaustion. This may be a result of neutrophils-induced apoptosis. There is upregulation of programmed death ligand 1 (PD-L1) and T cell immunoglobulin and mucin domain 3 (Tim-3), molecules that promotes the death of the target cell, which interacts with CD4^+^ and CD8^+^ lymphocytes to induce apoptosis (59, 60). SARS-CoV-2 may also infect T cells (61). Finally, SARS-CoV-2 may interfere with T cell expansion. MAP2K7 and SOS1, genes involved in T cell activation and function, may be downregulated in severe COVID-19 disease (62).

The subsequent alteration of the neutrophil-lymphocyte ratio is associated with increased nosocomial infection and mortality in severe sepsis (63).

Interestingly in sepsis, these pro- and anti-inflammatory phases appear to happen simultaneously as one response, and not as a distinct two-phase temporal relationship between pro- then anti-inflammatory immunity (55). Therefore, attempts to quantify patients into “hyperinflammatory” or “immunosuppressed” phenotypes may be an over-simplification of the host response, and a theranostic therapeutic approach may prove more difficult than initially proposed.

### Monocytes

In sepsis, there is downregulation of the human leukocyte antigen (HLA)-DR molecule on the monocyte, necessary for antigen presentation (64). There is also a reduction in LPS-induced TNF-α secretion from monocytes in sepsis, and these patients may benefit more from an immune adjuvant therapy such as G-CSF (65). This “immunoparalysis” correlates with increased risk of septic complications and death (66). The monocyte's lifespan, like the neutrophil's, is significantly prolonged in sepsis (67). Interestingly, in sepsis, hepatocytes release large amounts of high mobility group box 1 (HMGB-1) (a potent DAMP) which is transported to the cytoplasm of macrophages where it induces pyroptosis (a lytic form of cell death) resulting in depletion of the macrophage population, shock, multiple organ failure, and death (68). The phenotypic switch from M1 to anti-inflammatory M2 macrophages in sepsis also likely contributes to an immune suppressed state (69). Dendritic cells are also decreased in patients with septic shock, and their depletion is associated with increased mortality and health care associated infection (70, 71).

### Neutrophils

During septic shock, which may occur with COVID-19, neutrophils are systemically stimulated, which leads to impaired neutrophil migration to the infection focus. Bacterial components present in the blood activate TLRs expressed on neutrophils, leading to the upregulation of G protein-coupled receptor kinase 2 (GRK2), which induces internalisation of CXCR2 receptors on the neutrophil surface. Additionally, TLR activation induces the expression of TNF-α and iNOS (inducible nitric oxide synthase), the latter of which might also be activated by intracellular phosphatidylinositol-3-kinase (PI3K). Both TNF-α and NO (nitric oxide) can lead to upregulation of GRK2, exacerbating the downregulation of CXCR2 on the neutrophil surface. As a consequence, neutrophil trafficking is impaired in sepsis (72), reducing targeted microbial clearance. Furthermore, activation of TLRs also induces the expression of CCR2 on the surface of neutrophils. These activated neutrophils can migrate from inflamed tissues to other, non-infected, tissue and organ systems producing CCL2 (termed “reverse migration”), causing widespread host injury and organ dysfunction, potentially culminating in MODS (73, 74). It has been demonstrated that IL-33 can prevent the upregulation of GRK2 expression induced by TLR overactivation and consequently prevent the failure of neutrophil migration to the site of infection (73). This has not been described specifically in the novel disease process of COVID-19 but may outline the pathophysiologic mechanisms at play in this illness, and its propensity to induce distal organ injury.

Sepsis fundamentally alters the transcriptional profile of the innate immune system's key mediators—the macrophage and neutrophil. Upregulation of genes involved in inflammation and inhibition of apoptosis are seen in neutrophils in human subjects challenged with administration of endotoxins (75) as a model for bacterial sepsis. This response is similar to that seen in multi-trauma patients (76). In a non-septic patient, rapid apoptosis is seen within 24 h in 50% of neutrophils. A core difference in neutrophil activity consistently seen in sepsis is their ability to resist apoptosis with only 5–10% of neutrophils undergoing apoptosis in the first 24 h (77). This prolonged survival is mediated through alterations in gene expression with increases in key molecules like NF-κB (77), IL-1β (78), and PBEF/Nampt (79).

## Neutrophil Extracellular Traps

Neutrophil extracellular traps (NETs) were first described by Brinkmann in 2004 (80). NETs (Figure 1) are structures released from neutrophils comprising a core of chromatin DNA and histones, surrounded by specific antimicrobial proteins (lactoferrin, cathepsin G, defensins, LL-37, and bacterial permeability increasing protein), proteases (neutrophil elastase, proteinase-3, and gelatinase), and reactive oxygen species-generating enzymes (myeloperoxidase) (81). NETs are extremely efficient in pathogen trapping, killing, and prevention of pathogen dissemination. “NETosis,” the process of release of these extracellular traps, may be a new form of cell death that is different from apoptosis (3). CCL2, as well as recruiting immune cells, also signals for extracellular trap release (from neutrophils, mast cells, monocytes/macrophages, and eosinophils) (3), as does IL-6, CXCL-8, TNF-α, and IL-1β (associated with mast cell extracellular trap release). Activated endothelial cells may also encourage NETosis, which will ultimately kill these cells. “NETotic” neutrophils do not release apoptotic signals, do not undergo membrane blebbing, or perform nuclear chromatin condensation (3).

Dysregulated NETosis may lead to the development and exacerbation of several autoimmune and chronic infectious or inflammatory diseases (82). NETS have also been associated with multiple types of neoplastic processes (83). NETs can be released in a process of *suicidal* NETosis, where the neutrophil ruptures, or *vital* NETosis, where NETs are exocytosed from neutrophils in vesicles (84). In suicidal NETosis, several gramme-negative bacteria activate NADPH oxidase 2, which induces NETosis *via* reactive oxygen species production, while a NADPH-independent pathway for suicidal NETosis also exists, involving TLR-4-platelet-neutrophil interaction (85). This TLR-4-platelet-neutrophil interaction may be especially important in the pathogenesis of NET-induced “immunothrombosis.” Vital NETosis, however, also requires the presence of complement receptor-3 and TLR-2 (86). A recent paper highlights the key role that certain regulatory mitogen-activated protein kinases (MAPK), namely stress-activated protein kinase/c-Jun N-terminal Kinase (SAPK/JNK), play in regulating neutrophil survival. Specifically, a TLR-4/JNK activation axis exists, determining a neutrophil as NETotic or not (85).

Von Willebrand Factor (VWF) is exocytosed by activated endothelial cells onto their apical/luminal cell membrane, where the plasma glycoproteins then bind NETs *via* electrostatic bonds (84). VWF thrombogenic potential is tightly regulated in health by the metalloprotease ADAMTS13 (a disintegrin and metalloproteinase with thrombospondin type 1 motifs, member 13). NETs downregulate ADAMTS13 activity, promoting the formation, or inhibiting the degradation, of VWF multimers (84). NETs can be a significant source of enzymatic activity that may accelerate the formation of thrombi in blood vessels during infection (87, 88). As well as adhering NETs, VWF will also trap passing platelets and fibrinogen, allowing fibrin deposition and cross-linking, and ultimately vessel thrombosis (84). NETs also ultimately lead to alternative complement pathway activation, through neutrophil secretion of complement factor P, B, and C3, compounding the prothrombotic nature of NETs (Figure 1) (3, 89). This vicious cycle can potentially self-propagate unopposed in septic shock.

This process is supported by laboratory studies, where released NETs have been shown to disrupt alveolar epithelium and endothelium, and also degrade the thin alveolar basement membrane, culminating in epithelial necrosis, denudation of epithelial lining, vascular damage, pulmonary oedema, and haemorrhage in lethal influenza-infected mice (90). In humans, NETs have been shown to contribute to the development of ARDS in other severe viral respiratory infections, including H1N1 influenza (90). In COVID-19 pathogenesis, lung infection may accelerate local thromboembolic events, with neutrophils being a major contributor (91, 92). Mechanical ventilation may contribute however to an increased level of NETs markers in the alveoli of critically ill patients (93), compounding an already inflamed microenvironment. Another reason, perhaps, to be cautious regarding initiation of ventilation in COVID-19.

Extra-pulmonary injury from NETs has also been reported in COVID-19. Histone-induced tubular epithelial cell death results in acute kidney injury. Renal injury may be exacerbated with renal thrombosis due to NETs release. NETs may interact with hepatocytes *via* TLR2 and TLR4. Hepatocyte necrosis may occur secondary to damage from histones and C3a. Liver involvement also increases the propensity for thrombosis due to NETs (3).

## Stem Cell Therapies

Stem cells (regardless of age of donor or source tissue) are undifferentiated cells with capacity to self-renew and/or generate more than one differentiated functional daughter cell type. Mesenchymal stem cells (MSCs) are a specific population of stem cells with much therapeutic potential for sepsis. They are relatively immune privileged, avoiding the need for immunosuppression during use. MSCs may re-programme the immune system to reduce host tissue damage while preserving a strengthened immune response to microorganisms. They have also been shown to enhance tissue and endothelial repair following sepsis and have an extensive and growing safety profile in clinical trials (13).

Multiple pre-clinical septic animal models demonstrate the potential for MSCs therapy to reprogram neutrophil function to reduce host injury while maintaining bactericidal function (94, 95). MSCs reduce the infiltration of neutrophils to target organs, including liver, lung, intestine, and kidney, reducing injury and improving the function of these organs in preclinical sepsis models (94–99). MSCs also enhance neutrophil-mediated phagocytosis, making them more effective in the clearance of bacteria (95). Neutrophil depletion, using anti-Ly6G antibody, completely abrogated the protective effect of MSCs in systemic sepsis (95), highlighting the pivotal MSC-neutrophil interaction to the resolution of sepsis.

The Cellular Immunotherapy for Septic Shock (CISS) Trial, an open label phase 1 dose escalation trial for early septic shock, has led to the phase 2 CISS Trial, assessing safety and efficacy. Other trials include French (CHOCMSC [NCT02883803]) and Russian (100) studies. One clinical trial using cell-based therapies has been completed in COVID-19, using exosomes (extracellular vesicles derived from MSCs) (101). It demonstrated safety of MSC-derived exosome use in COVID-19, and potential as a therapeutic for this disease. At least 17 other clinical trials are in progress assessing MSCs in COVID-19-induced ARDS, as recently reviewed by Gonzalez et al. (102).

## Phenotypes

Phenotypic characterisation of illnesses may allow significant therapeutic advancement. In this regard, the identification of sub-phenotypes or “endotypes” within the sepsis population has been undertaken in patients with ARDS by Calfee et al. (103). A related approach, termed “Theranostics,” involves identifying biomarkers of therapeutic responsiveness. Man et al. (104) used this approach to identify potential subgroups of patients in the PROWESS-shock trial that may have benefited from activated Protein-C therapy (105). Similarly, Wong et al. (106) identified a paediatric septic shock subgroup that had a higher mortality from corticosteroid administration. Recently, Reddy et al. (107) published a review addressing subphenotypes in critical care, and how these can be translated into clinical practise.

IFN-γ and TNF-α drive a CXCL10/CCL2/macrophage phenotype seen in Crohns Disease and Rheumatoid Arthritis. Therefore, anti-TNF-α and janus kinase (JAK) inhibitors may be potentially successful therapeutic targets for COVID-19 (108). COVID-19 inflammatory phenotypes present in more severe illness progressing to mechanical ventilation have been described by Chua et al. (109). Several other authors have proposed clinical COVID-19 phenotypes (110–112), but the most extensive phenotypical characterisation to date is from Rodriguez et al. (113). Using unsupervised clustering analysis, Rodriguez characterised three novel clinical phenotypes. They associated the phenotypes with comorbidities and clinical outcome, using routinely available clinical and laboratory values, which may allow for easier and more economical future applicability of this model.

Septic patient populations can be divided into clinical or biomarker-driven subphenotypes, the latter focusing on more mechanistic and biologic categorisation. Translation of subphenotypes into clinical practise requires a better understanding of sepsis pathophysiology; how stable the subphenotypes are over time, how quickly, easily, and affordably we can diagnose them, and understanding the effect that multimorbidity has on these patient cohorts and their response to therapy. A theranostic approach may already have proven successful, by treating a specific subgroup of patients requiring oxygen in the first 24–48 h with anti-IL-6 therapy, leading to reduced mortality in a large trial by the REMAP-CAP group (33). However, this benefit in survival was not shared by the EMPACTA trial, investigating the same treatment with slightly different inclusion criteria (114).

## Potential Therapeutic Targets

Immune system disequilibrium is difficult to treat. To date, no specific anti-inflammatory treatment has been consistently successful in reducing morbidity or mortality in sepsis (115). Corticosteroids have shown much promise and act to inhibit NF-κB and AP-1 (116). Initially, low-dose corticosteroids were shown to reduce mortality in severe sepsis and septic shock by Annane et al. (117) but this was unable to be replicated in the larger CORTICUS randomised control trial, which showed no benefit (118). More specific blockade of proinflammatory molecules like TNF-α has also failed to show consistent success. A meta-analysis in 2013 showed a modest reduction in death in sepsis in patients given anti-TNF medications but concluded that larger trials with over 10,000 patients were needed to fully demonstrate this benefit (119). The benefit of immunomodulators in sepsis has been difficult to demonstrate for a variety of reasons, including difficulty with timing treatments, heterogeneity of the patient cohort, and variation of the underlying causes of sepsis (35). Many of these issues are not as prominent in COVID-19, with the disease course being more predictable, the typical patient cohort being slightly more homogenous, and the cause of the dysregulated inflammatory response being consistent. This may spell greater success for upcoming trials of immunomodulation in improving outcomes in COVID-19, many of which are discussed below.

With SARS-CoV-2 infection and immune system activation, many therapeutic targets exist. A theranostic approaches to finding a solution to the problems we have highlighted above may therefore succeed. Some old, yet rejuvenated, therapies, and some novel.

Approaches to altering the “Cytokine Release Syndrome” are 2-fold; block the action of a known cytokine propagator or increase the effects of an inflammatory down-regulator. Inhibition of the effects of IL-6, through blockade of its receptor (IL-6R) with Tocilizumab has received considerable attention, as IL-6 is thought to be a major initiator of the “cytokine storm” in COVID-19 (2). Initial trials in minority, non-ventilated populations failed (114), but more recent work by the REMAP-CAP (33) and RECOVERY (120) investigators in more critically ill patients has shown promise.

IL-17, produced by Th17 T-cells, is another proinflammatory cytokine (2). It is also produced by mast cells and NETs, and may play a role in thrombosis (3), as well as upregulating the production of other cytokines, most notably IL-6. Two monoclonal antibodies against IL-17, and one targeting the IL-17R have been successfully used in rheumatoid arthritis and psoriasis (2). The CXCL10-CXCR3 axis may also be a therapeutic target, especially blocking CXCL10 (eldelumab/MDX-1100) (121).

Corticosteroids have also been shown to reduce CXCL10 levels in COVID-19 (121), while separately, dexamethasone (122) and hydrocortisone (123) have been shown to reduce (rate ratio 0.83), and likely reduce (with a 93% probability) mortality, respectively. CXCL-14 potently inhibits epithelial cell chemotaxis, and is downregulated in COVID-19 allowing sustained and enhanced immune cell recruitment (3). This is, as yet an untargeted potential therapeutic.

Modulation of this overactive complement system has been attempted. Complement inhibition *via* AMY-101 (C3) or Eculizumab (C5) significantly reduced immune hyperactivation in severe COVID-19 (16). NLR was significantly altered by C3 inhibition, with reduced neutrophils and increased lymphocytes at day 7 compared to C5 inhibition. C3 inhibition resolved thrombocytopenia quicker than C5, and NETosis (*via* MPO-DNA levels) was reduced more profoundly, but not significantly, with C3 inhibition in both intubated and non-intubated patients. Ultimately, C3 inhibition may be better, preventing immune cell activation (*via* C3a–C3aR blockade), C3 opsonisation of epithelial or alveolar cells, and also the associated effects of C5 cleavage to C5a (C5a–C5aR inflammatory upregulation) and C5b (C5b–C9 MAC and cell lysis). Reduced neutrophil and T-cell recruitment *via* reduced C3a and C5a was also seen. C5a induction of monocytes and macrophages upregulates IL-6 production (3). Therefore, the viability of targeting the complement system seems more profitable than targeting a single cytokine or its receptor, due to the multi-layered effects of activating this system (C3, C5, inflammatory cell activation, and the MAC). More clinical trials may shed light on this [NCT04346797]. In a lung epithelial cell line study, ruxolitinib, a JAK1/2 inhibitor normalised interferon gene and complement gene signals induced by SARS-CoV-2, and reduced C3a production (19), showing potential to move into clinical trials. Another JAK1/2 inhibitor, Baricitinib, in combination with the antiviral agent Remdesivir, has shown benefit in hospitalised patients with COVID-19 (124).

Several therapeutics targeting innate immune cell recruitment, effector-memory T cells, or their phagocytic products are under assessment. A study assessing Vitamin C and its effects on COVID-19 patients by reducing neutrophil influx, activation and NET-associated alveolar capillary damage was abandoned due to difficulty in recruitment [NCT04264533]. The CXCR2 antagonists AZD5069 (blocks neutrophil trafficking but preserves neutrophil-mediated host immunity) and Danirixin and SCH527123 (both reduce neutrophil influx/migration) may be of benefit here (2). Neutrophil Elastase antagonists are either in clinical trial or approved for clinical use as treatments of ARDS pre-COVID-19 (2). Melatonin, a chronobiotic hormone, rejuvenates exhausted glutathione redox system in neutrophils during infection (125). Melatonin [NCT04409522], along with colchicine [NCT04350320] may also induce blockade of the inflammasome, offering other potential therapeutic targets in COVID-19.

Augmentation of the adaptive immune system is of particular interest in COVID-19, given the marked lymphopenia seen, potentially *via* upregulation of PD-L1 that induces lymphocyte apoptosis (60, 126). Its blockade may be a potential target in COVID-19 to improve outcomes (127) [NCT04356508, NCT04413838, and NCT04268537].

PAD (peptidylarginine deiminase) 4 inhibitors block NETs formation and release in murine sepsis models (128). Dipyridamole can inhibit NETs by activation of adenosine A_2A_ receptors (129), blocking adenosine reuptake and being a non-selective PDE4 inhibitor. Disulfaram as a therapy for COVID-19 is in clinical trial as a gasdermin D inhibitor, also inhibiting NETs formation [NCT04485130]. Hydroxychloroquine and Azithromycin can inhibit IL-1β and NET formation, but have not been shown to improve patient outcome in COVID-19 (130). The peptide-based agent Lupuzor/P140, trialled successfully in Systemic Lupus Erythematosus, may be of benefit in COVID-19 by blocking NET release but hasn't been trialled (131). Other NET-inhibitors include GSK-484 and BMS-P5, which have not been used *in vivo* as of yet (2).

Finally, dornase alfa (Pulmozyme, recombinant human deoxyribonuclease I) may improve ARDS in patients with severe COVID-19 through reduced mucus accumulation, lung injury, and improved gas exchange (132). However, the fragmented DNA may risk spreading inflammation beyond the area of viral invasion. Nine clinical trials are currently in progress for this therapeutic in COVID-19 (132).

## Conclusion

At the date of writing, global case incidence and related mortality of COVID-19 had surpassed 160 and 3.34 million, respectively. New, more transmissible strains of SARS-CoV-2 are now driving further waves of infection globally, and overwhelming health systems (133), with an inevitable surge in critically ill COVID-19 patients. With this, the vicious cycle of pulmonary epithelial cell infection and activation, cytokine and chemoattractant over-production, immune-cell recruitment, uncontrolled hyper-inflammation, and MODS continues. NETosis, while attempting to eradicate SARS-CoV-2, compounds this uncontrolled inflammation, with secondary “immunothrombosis” detrimental to the organ systems involved. Mechanical ventilation may compound this (93), and such support should be judiciously implemented. Emerging COVID-19 phenotypes may allow for more targeted therapy in the future. Currently, corticosteroids (122, 123), IL-6R antagonists (33, 120), and JAK inhibitors (124) are the only therapies showing promise for critically ill COVID-19 patients. Many hundreds of other clinical trials in COVID-19 maintain recruitment.

While vaccines against SARS-CoV-2 are being rolled out (134), further global pandemics are predicted (135). Future therapies against invasive pathogens revolve not only around their eradication but understanding better the deleterious effects they have on the human immune system, and how to regain and retain physiology over pathology. Perhaps trials using stem-cell-based therapies may shed some light.

## Author Contributions

CK conceived the presented idea and took lead in writing the manuscript. CK and MC wrote the manuscript in consultation with IM-L. All authors contributed to the article and approved the submitted version.

## Conflict of Interest

The authors declare that the research was conducted in the absence of any commercial or financial relationships that could be construed as a potential conflict of interest.

## Publisher's Note

All claims expressed in this article are solely those of the authors and do not necessarily represent those of their affiliated organizations, or those of the publisher, the editors and the reviewers. Any product that may be evaluated in this article, or claim that may be made by its manufacturer, is not guaranteed or endorsed by the publisher.
